# Hygroscopic properties modeling and thermodynamic analysis of a seamless popping capsule

**DOI:** 10.1038/s41598-025-15452-4

**Published:** 2025-08-17

**Authors:** Shuo Li, Hui Ge, Hongqiang Wang

**Affiliations:** Huabao Flavors and Fragrances Co., LTD, Shanghai, 201821 China

**Keywords:** Seamless capsules, Shelf life, Thermal stability, Isothermal adsorption curve, Moisture adsorption kinetics, Mathematical fitting, Biophysics, Drug discovery, Materials science, Mathematics and computing

## Abstract

This study investigated the thermal stability of seamless “popping” capsules at different storage temperatures to address the challenges of adhesion and texture changes affecting shelf life. Storage experiments at different temperatures identified optimal conditions for maintaining product integrity. Moisture adsorption behavior was analyzed using isothermal adsorption curves at 25 °C, 32 °C, and 37 °C, along with sorption kinetics at 68% relative humidity (RH). Capsules lost commercial viability at 37 °C and higher, and the moisture sorption isotherms followed Type II patterns. The Smith model best fit data at 25 °C and 37 °C (R^2^ > 0.98), while the GAB model was more suitable at 32 °C (R^2^ > 0.98). The storage humidity should not exceed 61% RH. The sorption process reached a state of saturation within two hours and followed first-order kinetics (R^2^ > 0.95). Thermodynamic analysis showed that sorption was enthalpy-driven and non-spontaneous, with an isokinetic temperature of 324.76 K and Gibbs free energy of 45.57 J/(mol·K). Attenuated total reflection Fourier transform infrared spectroscopy (ATR-FTIR) identified moisture-binding sites, and Scanning Electron Microscopy (SEM) revealed structural changes due to moisture exposure. These findings improve understanding of moisture sensitivity in seamless capsules and guide optimal storage conditions to preserve product quality.

## Introduction

Seamless capsules are widely used in various industries, including food, pharmaceuticals, and daily chemicals, due to their ability to encapsulate active ingredients, provide a unique texture, and enhance sensory experiences^1^. Seamless popping capsules, composed of a polymer-based gelatinous shell, burst upon biting or pressure, releasing their contents with a characteristic “popping” sensation. This sensory property, known as “popping taste”, is a key quality attribute that differentiates them from other encapsulated products^2^. In China, seamless popping capsules have gained popularity in the oral care market due to their novelty, portability, and quick effectiveness. The potential of seamless popping capsules in the functional food track is not only reflected in the novel and interesting product form, but also lies in its extension of the category attributes. Their ability to incorporate functional ingredients allows them to serve as a versatile alternative to traditional chewing gum, offering both a novel sensory experience and potential health benefits.

**Fig. 1:**
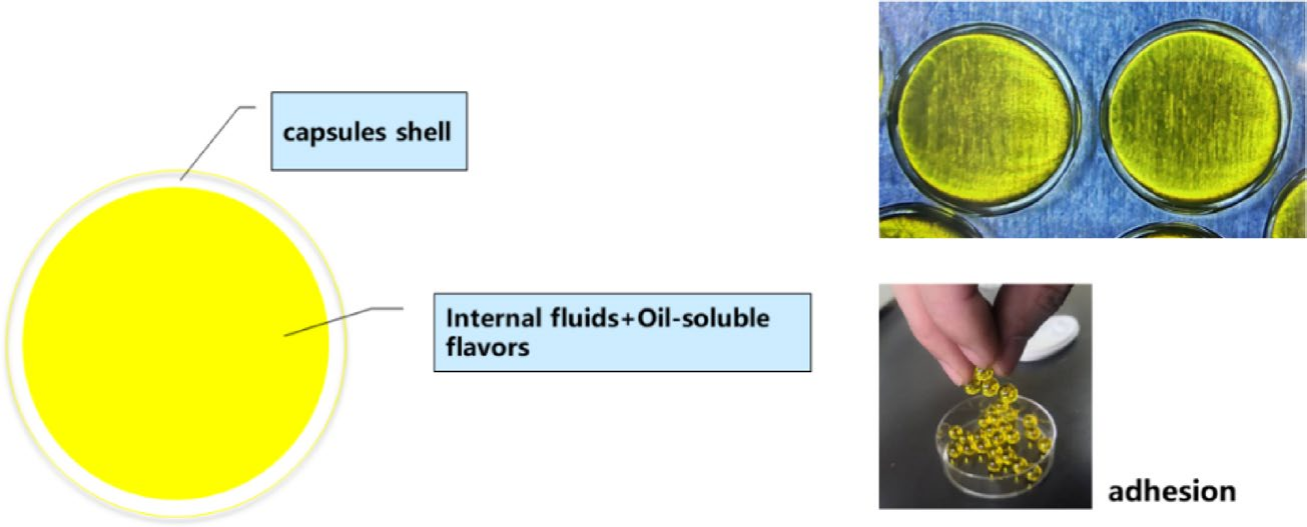
Brief introduction of seamless popping capsules.

However, seamless popping capsules are highly susceptible to moisture absorption due to their gelatin-based composition. Excess moisture leads to increased shell suppleness, reduced hardness and sensory experience. Additionally, elevated storage temperatures can accelerate the degradation of the capsule structure, resulting in the diminishing of the product quality. Despite their growing popularity, most research has focused on improving capsule formulation and manufacturing techniques ^3–6^, with limited attention given to their hygroscopic adhesion (Fig. 1) and storage stability.

To ensure product quality and longevity, it is essential to understand the moisture sorption characteristics of seamless popping capsules under different environmental conditions. Moisture sorption isotherms, which describe the relationship between the equilibrium moisture content (EMC) and the water activity (A_w_), provide valuable insights into moisture absorption^7^. These isotherms depend on the capsule’s chemical composition, structural properties, and physicochemical interactions with water^8^. This relationship is crucial for optimizing storage conditions and packaging strategies to prevent adhesion, structural degradation, and sensory alterations. Since gelatin-based materials exhibit strong hygroscopic properties, maintaining an optimal balance between temperature, humidity, and water activity is crucial for preventing these undesirable effects.

This study aims to assess the hygroscopic properties and thermodynamic characteristics of seamless popping capsules by evaluating their moisture sorption kinetics, isothermal adsorption process, and thermal stability under different temperature and humidity conditions. Through mathematical modeling, differential thermodynamic analysis, and structural characterization techniques such as ATR-FTIR and SEM, we seek to identify key factors influencing moisture adsorption and develop effective storage guidelines. The findings will provide theoretical guidance for improving the stability and shelf life of seamless popping capsules, ensuring their commercial viability and consumer appeal.

## Materials and methods

### Seamless popping capsules

**Fig. 2:**
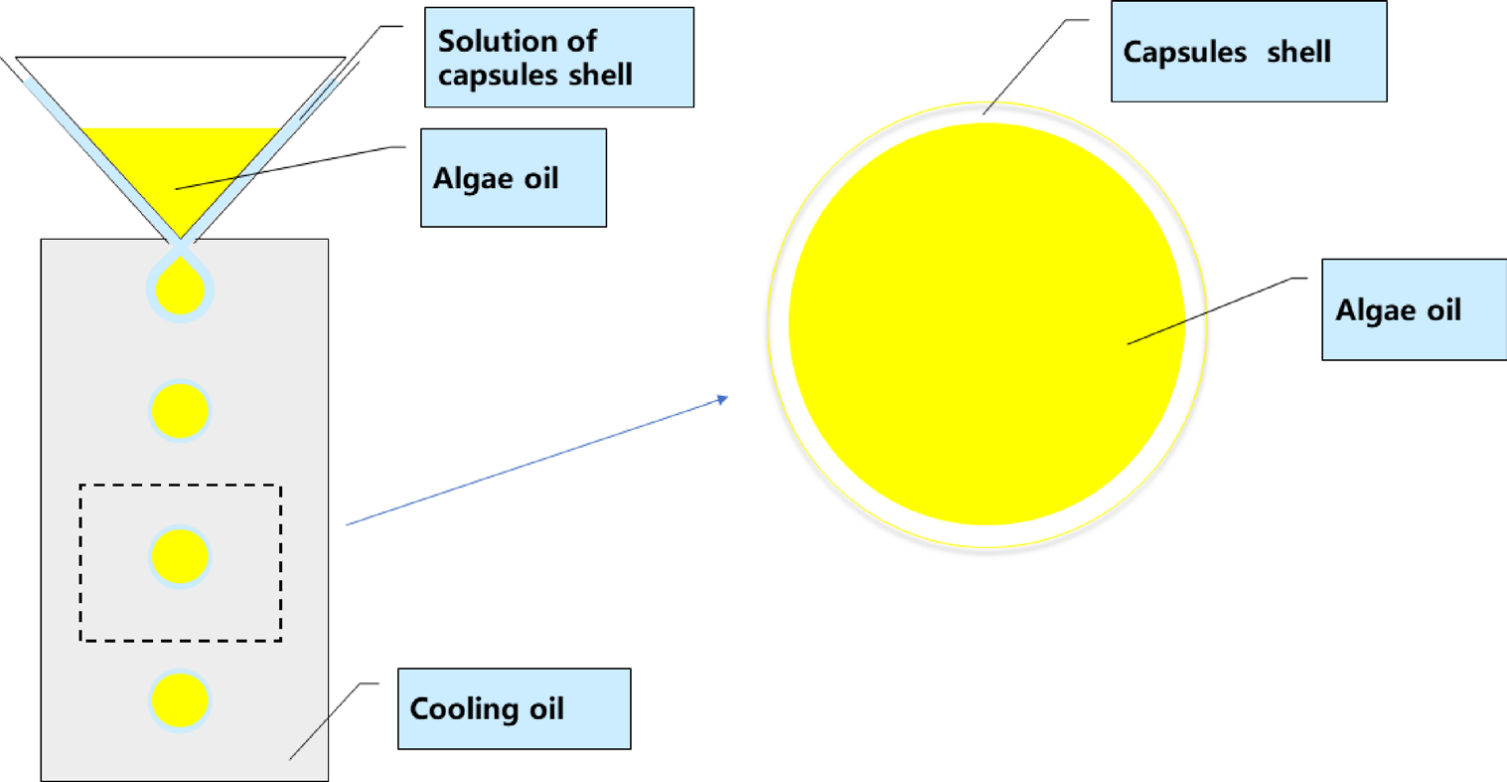
Production method of seamless popping capsules.

Seamless popping capsules were produced using a one-step dripping method (Fig. 2) in Jiangxi Xinhui Technology Co. (Jiangxi, China). Briefly, an oil-soluble flavor essence was dissolved in the internal liquid, which consisted of DHA algal oil. This was then encapsulated by edible polymer and dripped into a cooling liquid. Subsequently, the capsules or beads were collected, oil–removed, and dried to obtain the final products.

Reagents: potassium carbonate, sodium bromide, potassium iodide, sodium chloride, potassium chloride, sodium carbonate were purchased from Sinopharm (Shanghai, China).

### Mass fraction of the skin (M_fs_) of the seamless popping capsules

30 grains of seamless popping capsules were weighed randomly, and the average value was the mass of each capsule. The capsules were cut, and their contents were squeezed out. The edible polymer layers, containing gelatin, glycerin, and some polysaccharides, were weighed to obtain the mass of skin. The M_fs_ of seamless popping capsules was the skin mass divided by the mass of the capsules.

### Determination of the texture properties of the seamless popping capsules

The CT-3 texture meter (Ametek Brookfield Co. Middleboro, MA, USA) was used to analyze the texture of the capsules and record the hardness index^1^. Measurement parameters were as follows: load unit of grams, compression of 30%, down probe rate of 1.0 mm/s, waiting time of 3.0 s, trigger point load of 5.0 g, cycle once. Each sample was measured 5 times, and the results were averaged.

### Thermal stability storage experiment for seamless popping capsules.

Due to variations in the initial water activity of the seamless popping capsules over time, each capsule was equilibrated at 25 °C and 40% relative humidity (RH) for 24 h prior to experimentation. This conditioning ensured that the water activity was consistently maintained within the range of 0.40 ± 0.05. Then the seamless popping capsules were packed into boxes and vacuum-packed with double-layer composite aluminum foil paper to exclude the effect of moisture. The temperatures were in accordance with the actual needs of production, and were placed in the incubator at 25 °C, 32 °C, 37 °C, 45 °C, 52 °C and 56 °C, 40% RH respectively. The changes of the capsules were observed, and the thermal stability was investigated by the textural characterization.

### Isothermal adsorption curves determination of seamless popping capsules

Isothermal adsorption curves were determined referring to the method of Zhang et al^9^. Different saturated salt solutions can provide different relative humidity^10^.The saturated salt solution configured in advance was placed in a desiccator and a hygrometer was placed to monitor the equilibrium humidity in the desiccator. The desiccator was placed in a thermostatic incubator to ensure temperature equilibrium, establish a stable equilibrium vapor pressure, and maintain a consistent relative humidity. It was noted that there should be 10–20% precipitation in the saturated salt solution for the saturation during the test. The salt solutions and their corresponding humidity are shown in Table 1:

**Table 1:** Relative humidity of different solutions (Unit: %).

Solutions	Temperature ( °C)		
	25	32	37
K_2_CO_3_	45	46	45
KBr	62	61	58
KI	73	72	71
NaCl	79	77	79
KCl	88	84	87
Na_2_CO_3_	92	92	97

Seamless popping capsules were placed in Petri dishes and exposed to different saturated salt solutions to achieve equilibrium. The water activity (A_w_) of the samples equilibrated under each condition was then measured using an HD-7 moisture activity meter (Huake Instrumentation Co. Wuxi, Jiangsu, China), and the equilibrium moisture change rate (EMC)^11^ was calculated as the following equation:

1 $$EMC\% = \frac{{W_{1} - W_{0} }}{{W_{0} \times M_{{{\text{fs}}}} }} \times 100\%$$

where W_1_ is the mass of the capsules after moisture absorption, W_0_ is the mass of the capsules before moisture absorption and M_fs_ is the mass fraction of the skin. Before the experiment, the above final product version of capsules was placed at 25 °C and 37% RH, 32 °C and 37% RH, 37 °C and 37% RH conditions until equilibrium separately, as the initial state of the capsules under each temperature. The initial A_w_ values for the capsules were recorded as 0.3838 at 25 °C, 0.3874 at 32 °C, and 0.4156 at 37 °C, respectively.

### Isothermal adsorption model selection for seamless popping capsules

Based on five mathematical models^12–15^, GAB, Peleg, Smith, Halsey, and Henderson, the isothermal adsorption curves were nonlinearly fitted to select the best fitting model describing the equilibrium moisture process of the seamless popping capsules, using the coefficient of determination (R^2^) and the residual sum of squares (RSS) as the metrics (Table 2).

**Table 2:** Model isothermal adsorption curves.

Modelling	Displayed formula
GAB	$y=\frac{kcx\cdot {x}_{0}}{(1-kx)(1-kx+kcx)}$
Peleg	$y={ax}^{b}+{cx}^{d}$
Smith	$y=a+bln(1-x)$
Halsey	$y={(\frac{-a}{lnx})}^\frac{1}{b}$
Henderson	$y={(-\frac{1}{a}ln\left(1-x\right))}^\frac{1}{b}$

where *x* is the A_w_, *y* is the equilibrium moisture change rate (EMC), *k, c, a, b, d* are the parameters of each equation, and *x*_*0*_ is the initial moisture content.

### Hygroscopic kinetic curve determination of the seamless popping capsules

Due to the frequent occurrence of seamless popping capsule adhesion phenomena in summer, and considering that the average relative humidity in Shanghai, China, during summer ranges from 60 to 75%, a relative humidity of 68% was selected as the experimental condition.

A certain mass of the final product version of seamless popping capsule samples was weighed and placed in Petri dishes, then put in 25 °C and 68% RH, 32 °C and 68% RH and 37 °C and 68% RH conditions for storage separately. They were weighed every 0.5 h and the moisture gain rate (Mgr) in the seamless popping capsules skin was calculated as the following Eq. ^11^:

2 $$M_{gr} = \frac{{m_{1} - m_{0} }}{{m_{0} \times M_{{{\text{fs}}}} }} \times 100\%$$

where *m*_*1*_ is the mass of the capsules after moisture absorption, *m*_*0*_ is the mass of the capsules before moisture absorption, *M*_*fs*_ is the mass fraction of the skin. Moisture adsorption kinetics was plotted with *M*_*gr*_ as the vertical coordinate and time as the horizontal coordinate.

### Selection of hygroscopic kinetic model for seamless popping capsules

As shown in Table 3, Four hygroscopic kinetic models were used were utilized to describe the hygroscopic kinetic processes of seamless popping capsules at 25 °C and 68% RH, 32 °C and 68% RH, and 37 °C and 68% RH, and curve fitting was performed^16–19^. The coefficient of determination (R^2^) and residual sum of squares (RSS) were used as indicators to determine the optimal fitting model for the hygroscopic kinetic curves of capsules.

**Table 3:** Kinetic models of hygroscopicity.

Modelling	Displayed formula
Zero class	$y=a+bx$
First-order kinetics	$y=a(1-{e}^{-bx})$
Second-order kinetics	$y=\frac{{ab}^{2}\text{x}}{1+abx}$
Biexponential model	$y={A}_{1}{e}^{-\frac{x}{c}}+{A}_{2}{e}^{-\frac{x}{d}}+ {y}_{0}$

where *y* is the M_gr_ of gelatinized skin, *x* is time, and *y*_*0*_, *A*_*1*_, *A*_*2*_, *a*, *b*, *c* and *d* are the corresponding model parameters.

### Scanning electron microscopy

The microscopic morphology of the seamless popping capsules skin after reaching hygroscopic equilibrium in section “Hygroscopic kinetic curve determination of the seamless popping capsules” was observed with a Scanning Electron Microscope (Hitachi S3400, Tokyo, Japan). Samples were embrittled with liquid nitrogen and then placed in ion sputtering unit and gold sprayed at 15 mA for 60 s to improve their conductivity^3^. The surface structure and cross-sectional of the seamless popping capsules skin was observed at an accelerating voltage of 15 kV by 2 kX. 15 kX and 30 kX.

### Attenuated total reflectance-Fourier transform infrared spectroscopy (ATR-FTIR)

An ATR-FTIR (Thermo Scientific, Nicolet iS10, Waltham, MA, USA) was used to determine functional groups and chemical bonds in the seamless popping capsule shells with a resolution of 4 cm^−1^, a wave number range of 50–4000 cm^−1^, and 32 scans at room temperature (25 °C)^20^.

### Differential thermodynamic analysis

The hygroscopic capacity of seamless popping capsules was computed by differential thermodynamic analysis. Gibbs free energy (*ΔG*), the net isosteric heat (*q*_*st*_) and entropy of adsorption (*ΔS*_*d*_) were computed from the water vapor adsorption isotherms experimentally obtained at 25 °C, 32 °C,37 °C using a differential strategy^12,20–22^.

#### Determination of the Gibbs free energy

The change in Gibbs free energy (*ΔG/*(J/mol)) was used to indicate the affinity of the adsorbed substrate for water, designating whether water adsorption was a spontaneous process, with the following equation:

3 $$\Delta G = - RT\ln A_{{\text{w}}}$$

where *R* is the gas constant, 8.314 J/(mol·K),*T* is the absolute temperature (K), and *A*_*w*_ is the water activity.

#### Net isosteric heat determination of adsorption

The net isosteric heat, or enthalpy of adsorption (*q*_*st*_ (J/mol)) reflected the force strength between the water molecules and the adsorption sites of the solid matrix, and the Clausius–Clapeyron equation was used for calculating the *q*_*st*_ of adsorption, as in the following equation:

4 $$\frac{{\partial \ln A_{w} }}{\partial 1/T}|_{{x = x_{EMC} }} = - \frac{{q_{st} }}{R}$$

#### Differential entropy determination

The differential entropy (*ΔS*_*d*_ (J/(mol·K))) is related to the attractive and repulsive forces between the water molecules and the material and is proportional to the number of moisture adsorption sites of the material, which can be calculated by the Gibbs–Helmholtz equation as follows:

5 $$\Delta {S}_{d}=\frac{{q}_{st}-\Delta G}{T}$$

Substituting the Gibbs free energy equation (Eq. (3)) into the above equation yields the relationship between the net isosteric heat (*q*_*st*_) of adsorption and the differential entropy (ΔS_d_), as following equation:

6 $$\ln Aw|_{{x = x_{EMC} }} = - \frac{{q_{st} }}{RT} + \frac{{\Delta S_{d} }}{R}$$

from which it follows that the magnitude of *ΔS*_*d*_ is approximately equal to the intercept of the linear fitting between *ln A*_*w*_ and *1/T* at a constant equilibrium moisture content. Where *x* is the moisture content (%) at which the *EMC* is reached.

#### Spreading pressure

According to Tanuja and Ravindra^22^, the spreading pressure (*ф*), or surface potential, is the surface excess free energy and can be defined as the difference between the chemical potential of pure adsorbent and that of the adsorbent in sorption, resulting in an increase in the surface tension of bare sorption sites because of the sorbed molecules. The calculation method was derived from the following Eq. ^23^:

7 $$\phi = \frac{{K_{B} T}}{{A_{M} }}\ln (\frac{{1 - k_{g} \cdot A_{w} + k_{g} c_{g} \cdot A_{w} }}{{1 - k_{g} \cdot A_{w} }})$$

The parameters of the GAB model at 25 °C, 32 °C, and 37 °C were used for computation. Here, *K*_*B*_ is the Boltzmann’s constant (1.38 × 10^–23^ J/K) and *A*_*M*_ is the area of a water molecule (1.06 × 10^–19^ m^2^), and *k*_*g*_ and *c*_*g*_ are the GAB constants.

#### Enthalpy–entropy compensation theory

The Enthalpy–Entropy compensation theory was used to assess the physicochemical phenomena occurring during moisture adsorption, which stated that the *q*_*st*_ has the following linear relationship with *ΔS*_*d*_:

8 $${q}_{st} ={T}_{\beta }\cdot\Delta {S}_{d}+\Delta {G}_{\beta }$$

where *T*_*β*_ denotes the isokinetic temperature (K) and ΔG_*β*_ is the Gibbs free energy at the isokinetic temperature (J/(mol·K)).

Krug et al^24^ introduced the concept of mean harmonic temperature *T*_*hm*_ (K), which was calculated as the following equation:

9 $${T}_{hm} =\frac{N}{\sum_{i=1}^{N}(\frac{1}{{T}_{i}})}$$

where *N* is the total number of isotherms and *Ti* (K) is the set temperature of the isothermal adsorption experiment. They proposed that the theory of entropy–enthalpy compensation is applicable when the characteristic temperature of moisture adsorption (*T*_*hm*_) differs from the isokinetic temperature (*T*_*β*_). Specifically, when *T*_*hm*_ is lower than *T*_*β*_, the adsorption process is governed by enthalpic interactions, whereas when *T*_*hm*_ exceeds *T*_*β*_, the process is predominantly entropy-driven.

#### Statistical analysis

Results were shown as mean values ± standard deviation. The one-way analysis of variance (ANOVA) with Duncan’s multiple range test was used to analyze the data. SPSS version 21.0 software (SPSS, Chicago, IL, USA) was used for statistical analysis. *P* < 0.05 was considered statistically significant. The data were fitted using ORIGIN 2020Pro (Originlab, Northampton, Massachusetts, USA) and MATLAB R2023 (MathWorks, Natick, Massachusetts, USA).

## Results and discussion

### Changes in the hardness of seamless popping capsules during storage

**Fig. 3:**
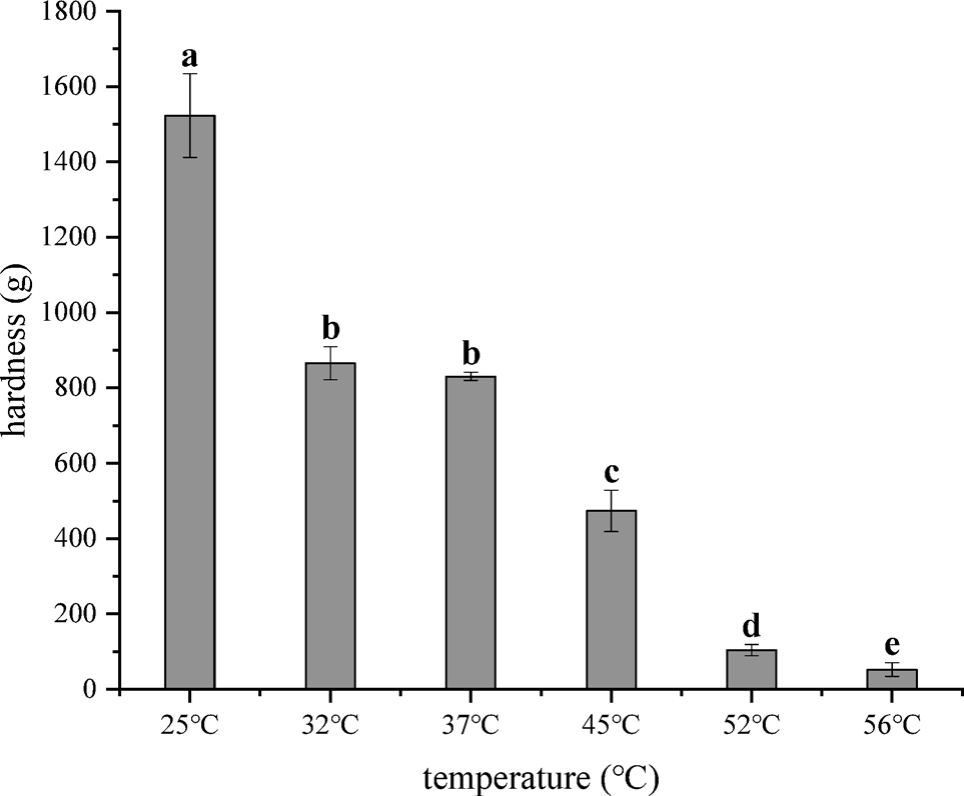
Changes in the hardness of seamless popping capsules at different temperatures (40% RH). Different letters indicate significant differences in results, *P* < 0.05.

As illustrated in Fig. 3, the hardness of the capsules exhibited distinct temperature-dependent changes. An initial sharp decline was observed as the temperature increased from 25  to 32 °C, with hardness decreasing from 1500 to 900 g. Between 32  and 37 °C, the reduction in hardness was minimal. However, a second notable decrease occurred from 37C to 45 °C, where the hardness dropped from 850 to 500 g. A further substantial decline was recorded between 45 and 52  °C, reaching a final value of 100 g. When the temperature was 32 °C, the first significant change in the texture of the capsules occurred, indicating that the temperature 32 °C could affect the steady state of the seamless popping capsules, and there was a further drop (< 600 g, without commercial value) at 45 °C Therefore, three temperature gradients were selected for the hygroscopicity test. As the samples were sealed using a double layer of aluminum foil packaging to minimize water vapor exchange between the seamless popping capsules and the external environment, it can be assumed that, under constant moisture content, the water activity of the capsule skin increased with rising temperature. When the capsule skin reached its thermal transition temperature, a reduction in hardness was observed^20^.

### Hygroscopic isotherm models for the seamless popping capsules

Water vapor adsorption is a physical process ^23^ and is commonly employed in evaluating the moisture adsorption capacity of food products, such as starch ^9^. The results of the hygroscopic isotherm model fitting are presented in Table 4. In this study, the water activity began at 0.43. Since previous studies have shown that the BET model provides a better fit within the water activity range of 0–0.45, it was not selected for use in this experiment^25^. Admittedly, higher R^2^ and lower RSS indicate a better fit to the model equation. Among the five commonly used adsorption isotherm models, the most appropriate model for plotting isothermal adsorption curves was the Smith model for 25 °C samples (higher R^2^) and 37 °C samples, the most suitable model for 32 °C was the GAB model. The specific model fitting parameters are shown in Table 5.

**Table 4:** Model parameters of isothermal adsorption curves.

Sample	25 °C	32 °C	37 °C
Model form	$y=a+bln(1-x)$	$y=\frac{kcx\cdot {x}_{0}}{(1-kx)(1-kx+kcx)}$	$y=a+bln(1-x)$
Model	Smith	GAB	Smith
x_o_	–	177.03	–
a	− 8.05	–	− 2.63
b	− 17.01	–	− 7.98
k	–	0.89	–
c	–	13.17	–

**Table 5:** Results of the hygroscopic isotherm model fitting.

Sample	Model	R^2^	RSS
25℃	GAB	0.9892	0.9633
	Peleg	0.9882	1.7610
	Smith	0.9965	1.0379
	Halsey	0.9719	8.3792
	Henderson	0.8692	39.0666
32℃	GAB	0.9925	0.9956
	Peleg	0.9159	7.5203
	Smith	0.9608	7.0124
	Halsey	0.7553	43.7983
	Henderson	0.5203	85.8499
37℃	GAB	0.9782	11.6500
	Peleg	0.9626	14.0896
	Smith	0.9827	13.0120
	Halsey	0.7263	206.2091
	Henderson	0.9183	61.5527

**Fig. 4:**
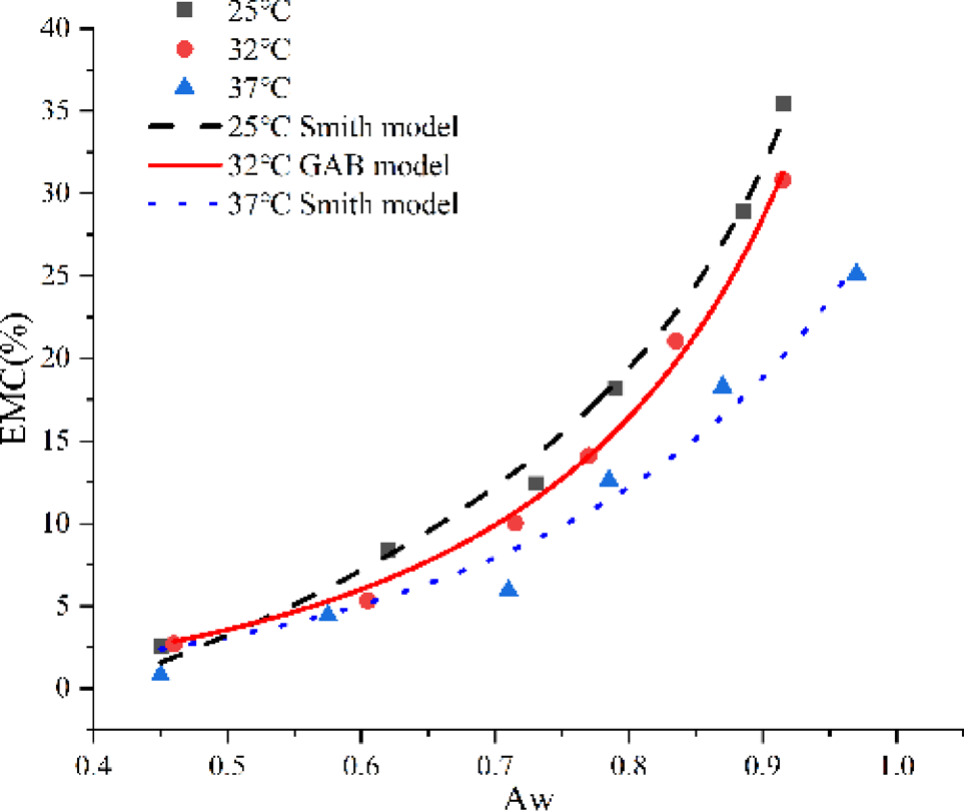
Isothermal adsorption curves of seamless popping capsule at 25 °C 32 °C and 37 °C

**Fig. 5:**
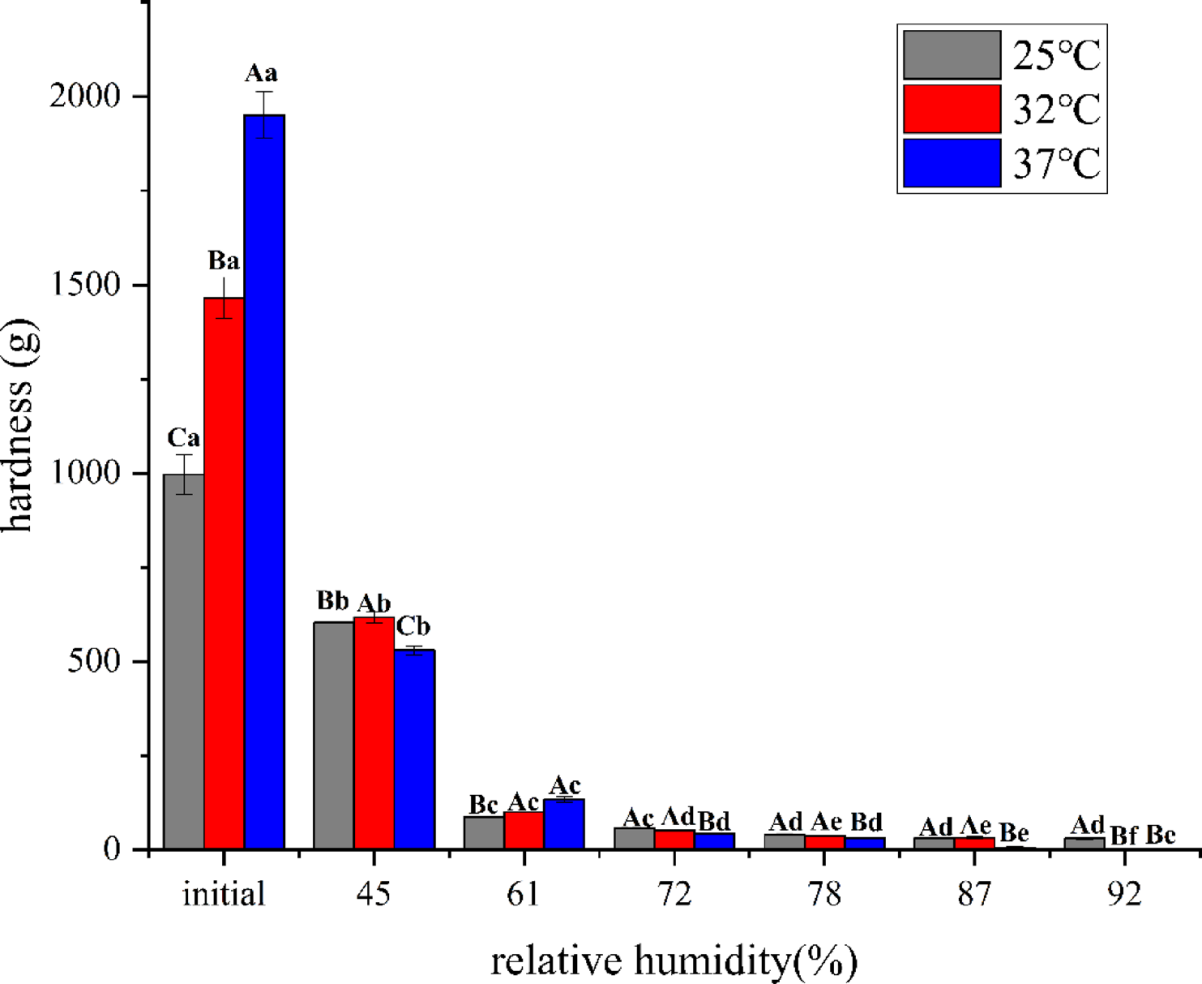
Hardness of seamless popping capsules after equilibrium at different humidity. (Upper case letters are significant differences between samples of approximating relative humidity at different temperatures, lower case letters are significant differences between samples of the same temperature).

As shown in Fig. 4, the isothermal adsorption curve of the algae oil seamless popping capsule resembled the S-shaped isotherm^26^, which is characteristic of biopolymers such as chia seed mucus, chitosan^13^. It can be observed that when the relative humidity in the environment is about 43%, all the bursting beads begin to exhibit hygroscopic behavior, corresponding to a decrease in hardness in the texture data (Fig. 5). The hardness of the popping capsules is highly sensitive to moisture, which is due to the presence of hydrophilic polymers in the capsule skin. When the water activity up to 0.60, the hardness of the capsules under various temperatures underwent an unacceptable transformation. Therefore, for this seamless popping capsules, special attention should be paid to minimizing the relative humidity of the storage environment, particularly when the temperature is below 37 °C. This imposed stricter requirements on product packaging and moisture control strategies. Temperature affects the water activity of the seamless popping capsules, as temperature rises, the water molecules in the capsules would enhance the water activity, affecting the equilibrium moisture content adsorption of isotherm, causing the isothermal adsorption curve to shift downward. Moreover, on the same adsorption curve, it can be observed that under certain humidity conditions, seamless popping capsules reach adsorption equilibrium. However, when transferred to a higher humidity environment, the capsules continued to absorb moisture until a new equilibrium was established. This behavior indicates the absence of a fixed adsorption saturation point, characteristic of a Type II isotherm. Combining with the changes in the textural properties of seamless popping capsules during thermal storage, it is possible to derive a safe storage environment for the capsules, including ambient temperature and humidity. Similarly, previous studies have determined the safe moisture content of animal feed by analyzing its hygroscopic behavior^27^. Based on these findings, it can be concluded that the optimal relative humidity range for the safe storage of seamless popping capsules lies between 43 and 61%. Additionally, maintaining an ambient temperature below 37 °C is recommended to preserve product stability under these conditions.

### Moisture absorption kinetics of the seamless popping capsules

**Fig. 6:**
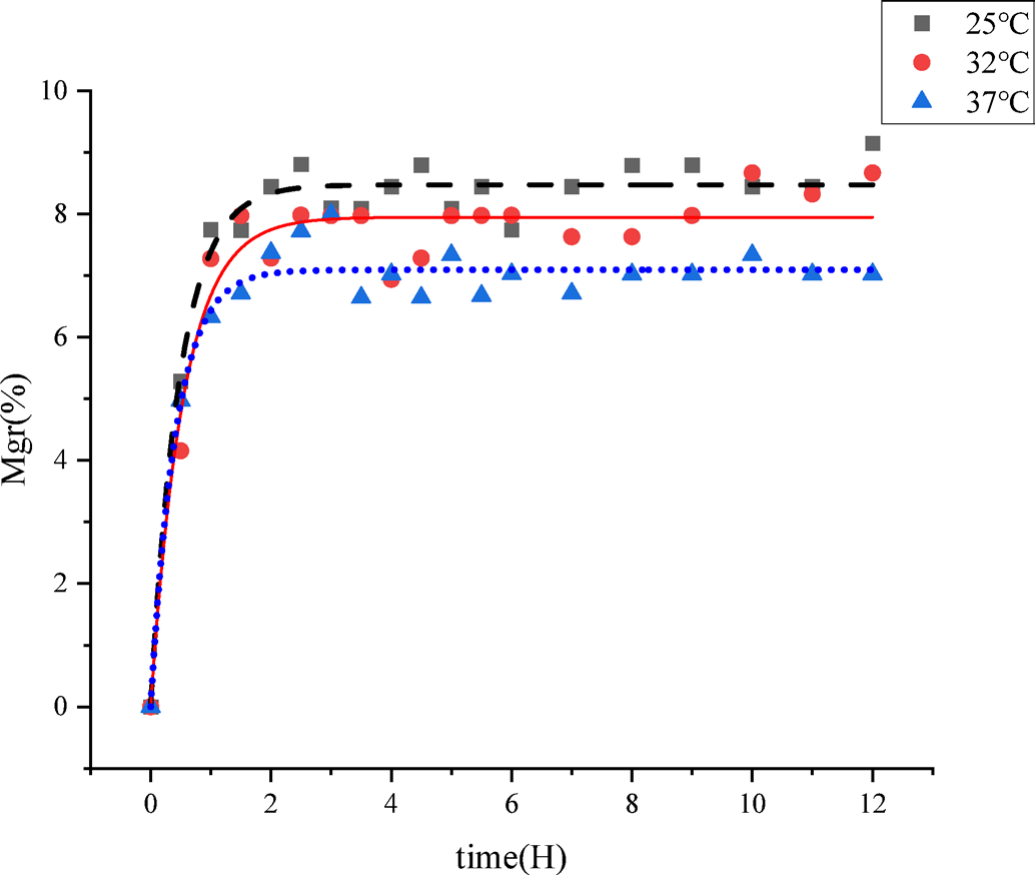
Moisture absorption kinetic curves of seamless popping capsules (68% RH).

The Table 6 represents the effect of fitting each model for the moisture absorption kinetics of the shell of seamless popping capsules. The hygroscopic properties were determined under steady state conditions^28^. The results showed that the moisture absorption quantity of the seamless popping capsules shell was high at 68% RH, and the moisture absorption was saturated within 2 h. Previous studies have reported that dry, porous surfaces are nearly impermeable to water droplets at room temperature and can only be penetrated when either the surface temperature rises or the surface is pre-wetted^29^. This phenomenon may also account for the moisture adsorption behavior observed on the capsule shell surface during the initial two hours. Pre-wetting of the capsule skin due to moisture adhesion led to water penetration and a rapid increase in moisture absorption. Thomas Busser et al^30^ highlighted that in the standard methodology for generating moisture absorption curves, discrepancies often arise between numerical predictions and experimental data. These differences are primarily attributed to the fact that such models are based on steady-state conditions, representing the system after moisture absorption has reached equilibrium (Fig. 6). The zero- class kinetic curve apparently didn’t fit and was excluded, the first-order kinetic fitting effect was the best, followed by the biexponential model. Several researchers have noted that the biexponential model had a better fitting effect^16,18^. However, the biexponential model in this study had more coefficients, which was not favorable for further analysis, and the fit was not as good as that of the first-class kinetic model (smaller R^2^ value). Therefore, the first-class kinetic model was preferred for the hygroscopic kinetics of seamless popping capsule shell. In the results of Table 7, value *a* and *b* in the first-order kinetic model have physical significance, with value *a* indicating the amount of moisture increase when moisture adsorption is saturated, is the macroscopic value, corresponding to *X*_*0*_ in the isothermal adsorption GAB model is the amount of water change at saturation of water adsorption by monolayer capsule shell molecules and value *b* indicating the rate of moisture absorption. It has been reported that the moisture absorption equilibrium of hydrolyzed peptide powders was a simultaneous process of moisture absorption and desorption, and water was transited from free water state to fixed water state, and finally to bound water state^11,31^. Whether similar phenomena occur in seamless popping capsules remains to be investigated; however, this will be addressed in the following sections. The secondary kinetic model is a model proposed under the assumption that chemical absorption occurs during the absorption process, and there is sharing and transfer of electrons^31^. As it was not fitted as well as the primary kinetic model, we infer that there was no chemical absorption during the moisture adsorption process of the capsule.

**Table 6:** Effect of fitting each model for seamless popping capsules.

Model		Zero class	First-order kinetics	Second-order kinetics	Biexponential model
Model form		$y=a+bx$	$y=a(1-{e}^{-bx})$	$y=\frac{{ab}^{2}\text{x}}{1+abx}$	$y={A}_{1}{e}^{-\frac{x}{c}}+{A}_{2}{e}^{-\frac{x}{d}}+ {y}_{0}$
25 °C and 68%RH	R^2^	0.2242	0.9700	0.9583	0.9636
	RSS	55.4824	2.1470	2.9799	2.1464
32 °C and 68%RH	R^2^	0.2706	0.9712	0.7571	0.9275
	RSS	49.5353	2.1470	16.4987	4.0576
37 °C and 68%RH	R^2^	0.1183	0.9570	0.9308	0.9439
	RSS	43.5145	2.2805	3.4158	2.2804

**Table 7:** Parameters of kinetic equation for moisture absorption of seamless popping capsules under different storage conditions.

Model form	Storage conditions	a	b
$y=a(1-{e}^{-bx})$	25 °C and 68%RH	8.48 ± 0.09c	2.05 ± 0.19ab
	32 °C and 68%RH	7.95 ± 0.13b	1.84 ± 0.24b
	37 °C and 68%RH	7.13 ± 0.09a	2.40 ± 0.31a

Annotation: Different letters indicate significant differences in data between the same columns.

**Fig. 7:**
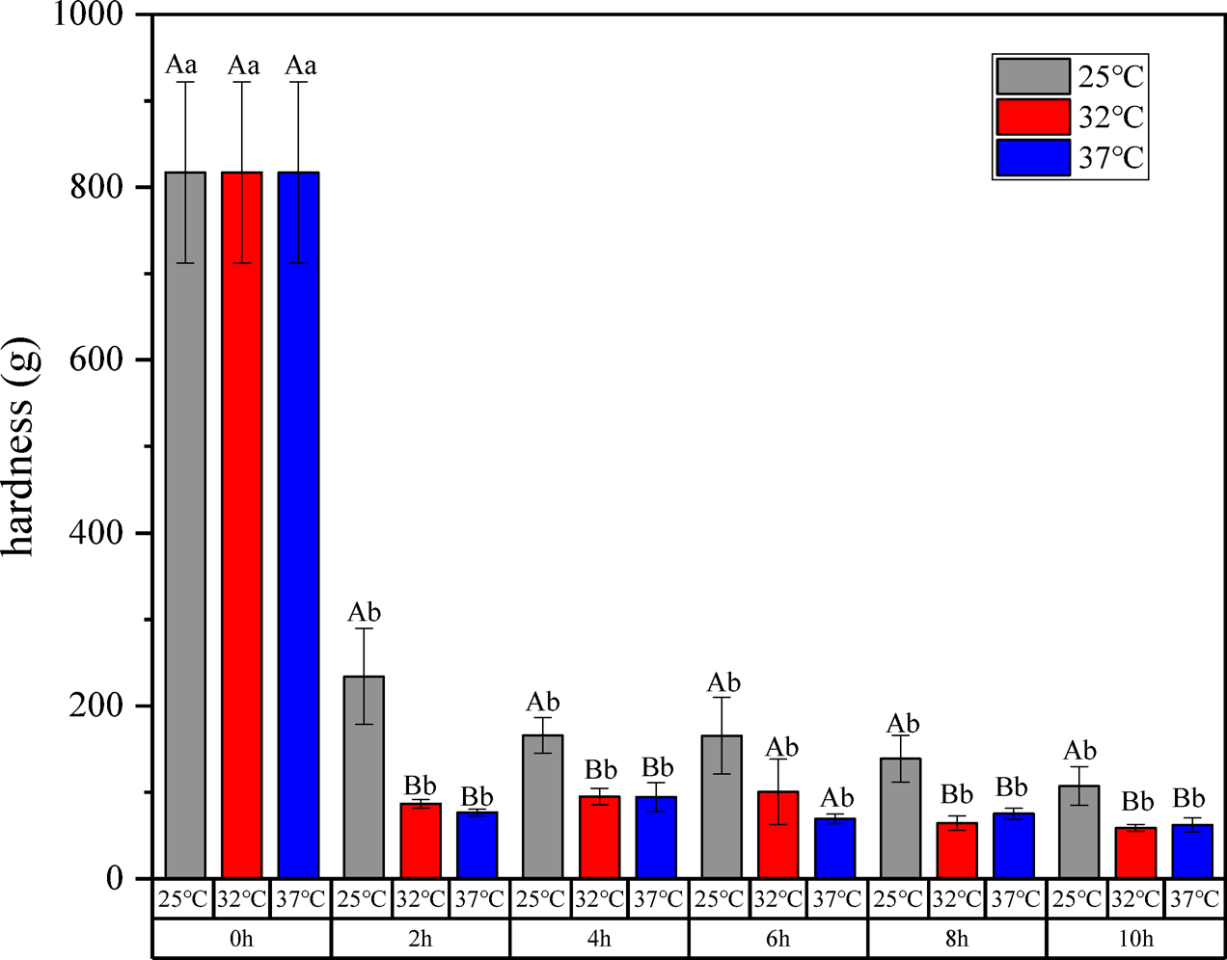
Texture changes in the hygroscopic process of seamless popping capsules (68% RH). Upper case letters are significant differences between samples at different temperatures, lower case letters are significant differences between samples of the same temperature.

The Fig. 7 shows the texture change of the seamless popping capsules under the moisture absorption process of the seamless popping capsules at 68 RH%. Hardness is the ideal quantitative index of the popping taste of seamless popping capsules, and high popping taste is one of the most important guidance for the high-quality seamless popping capsules^1,2^. As can be seen from the figure, the hardness of the seamless popping capsules in the first 2 h decreased significantly, and then tended to equilibrate, which is consistent with the trend of moisture absorption of the capsules skin. The presence of water molecules enhances the flexibility of the polymer molecule chain segments, resulting in the softening of seamless popping capsules hygroscopic.

### ATR-FTIR analysis

**Fig. 8:**
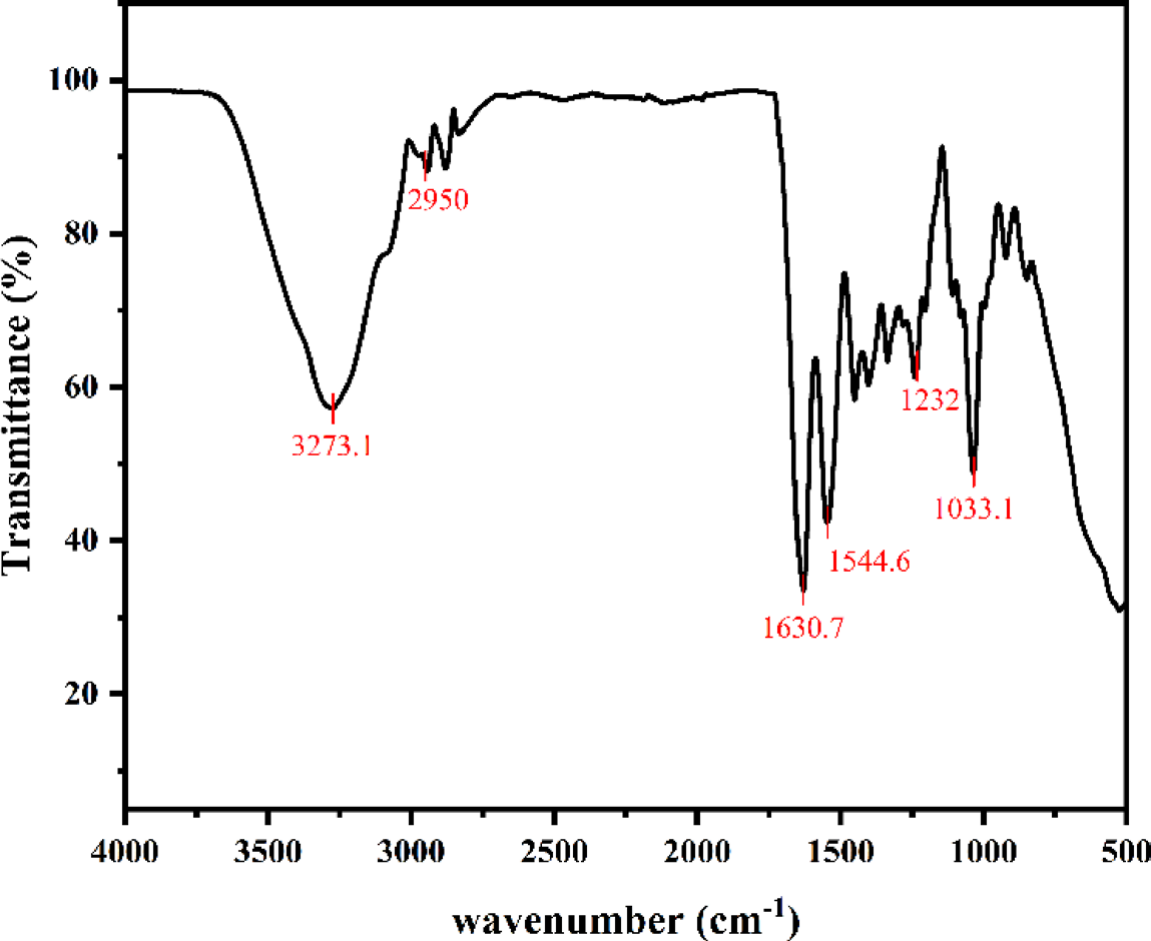
Infrared spectra of seamless popping capsule shells.

Figure 8 showed the ATR-FTIR spectra of the capsule shell. The specific functional groups in capsule shell were a broad peak at 3273 cm^−1^ (O–H stretching vibration), 1630 cm^−1^ (C = O stretching of amide-I) and 1544 cm^−1^ (N–H stretching of amide-II). The peaks around 2950 cm^−1^ were related to the stretching vibration of the C–H contraction vibration in the methyl group. The absorption peak at 1033 cm^−1^ was associated with stretching vibrations within the C–H plane, and that at 1232 cm^−1^ was a functional group of secondary alcohols. These were binding sites associated with water absorption, what’s more, these were consistent with the report by Li et al. regarding gelatin^32^, which indicated that the main material of the seamless popping capsule shell was gelatin, and glycerol might be added to enhance the hygroscopicity of the capsule shell.

### Surface morphology of the seamless popping capsules

**Fig. 9:**
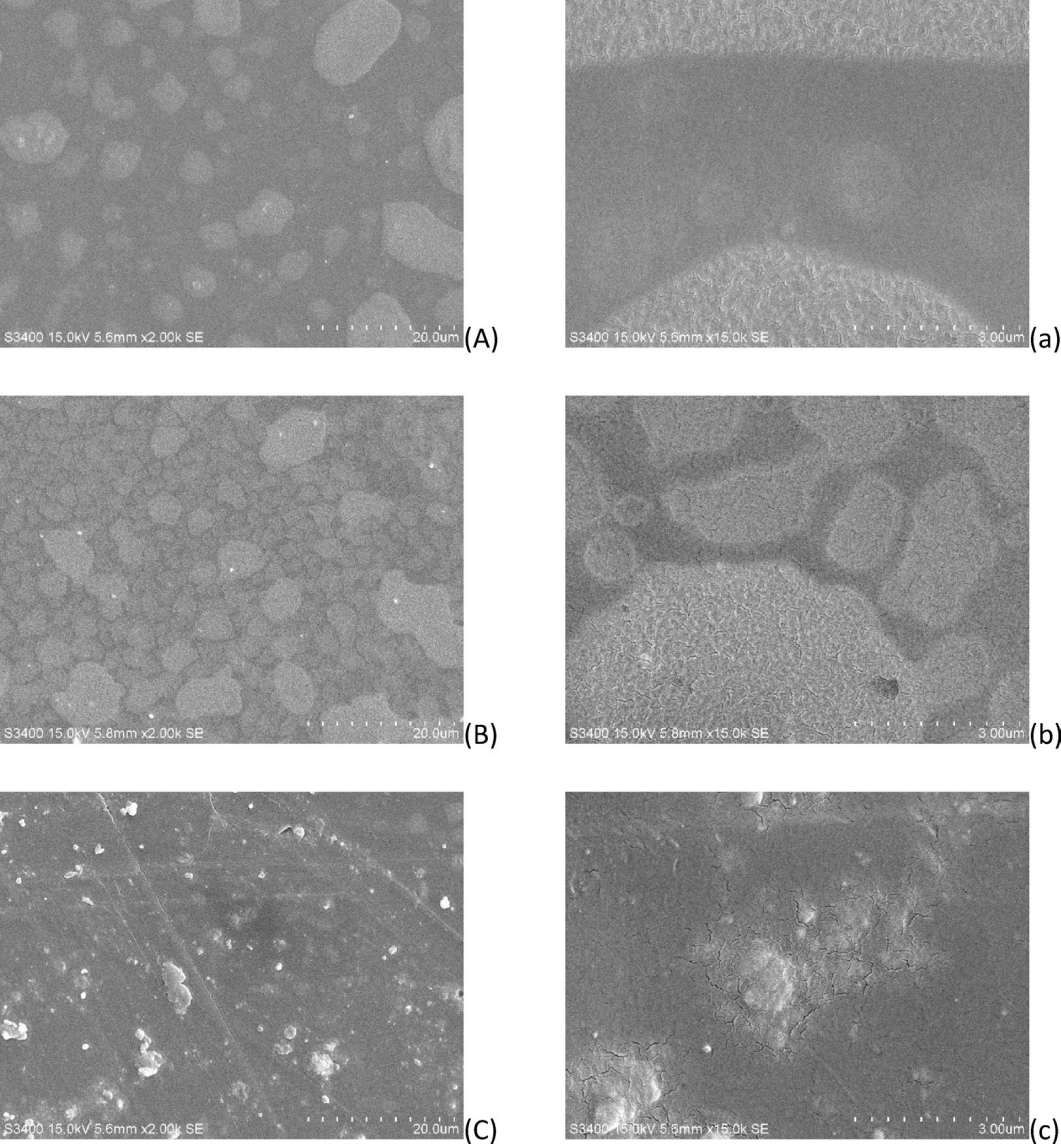
Surface microstructure of seamless popping capsule skins. (**A**) and a: 25 °C, 2kX and 15kX; (**B**) and b, 32 °C, 2kX and 15kX; (**C**) and c: 37 °C, 2kX and 15kX.

**Fig. 10:**
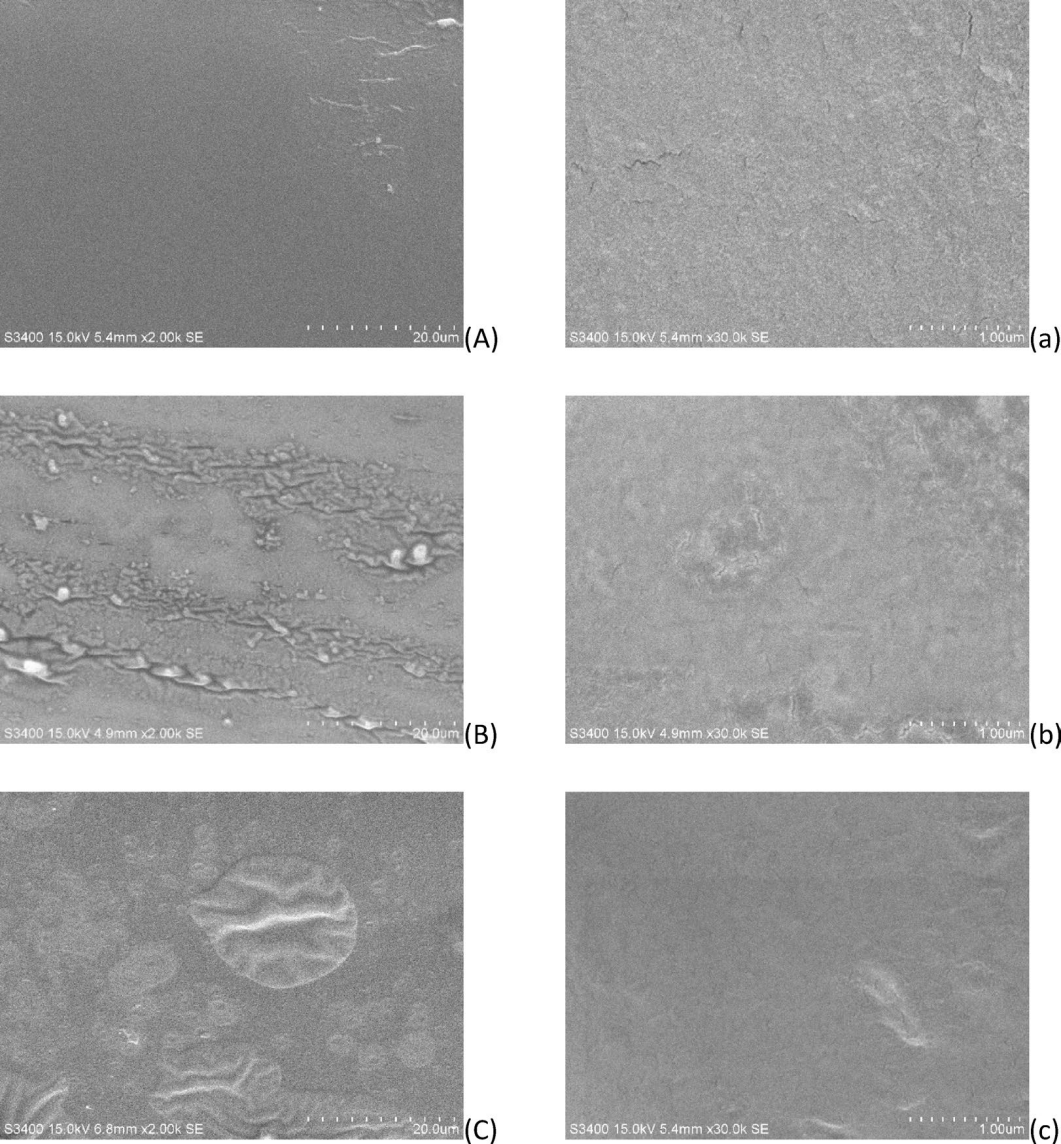
Microstructure of cross-sectioned surfaces of seamless popping capsule skins. (**A**) and a: 25 °C, 2kX and 30kX; (**B**) and b, 32 °C, 2kX and 30kX; (**C**) and c: 37 °C, 2kX and 30kX.

As shown in the 2kX of Fig. 9, there were many small patches on the surface of the samples at 25 °C and 32 °C, while for the sample at 37 °C, there were many small protruding flakes attached on the surface. From the images of 15kX, fine cracks were seen on the patches, and the surface of the sample at 37 °C seemed to be rougher, and have longer and wider cracks. From those topographical and morphological characteristics, it could be speculated that more cracking and local agglomeration occurred on the surface at high temperatures, decreasing the structural strength of the capsules. Another explanation was that the capsule shell became more porous as the surface temperature rose, so that the moisture could enter the capsule shell to reduce its hardness. Zhang et al^29^ found that the water drop barely penetrated on the dry porous surface at ambient temperature, but penetrated when the surface temperature was raised or the surface was pre-wetted. From the results of the cross-section (Fig. 10), under the same magnification, that is, the same field of view, samples at 25 °C cracked less and smooth, the structure of the cross-section at 32 °C occurred more agglomeration, wrinkles and cracks, which would reduce the structure strength of the seamless popping capsules. Another undesirable phenomenon occurred in the foam at 37 °C, which might be caused by the penetration of moisture, and further reduced the seamless popping capsules’ oral popping sense. The uneven distribution of macromolecules (agglomeration in Fig. 10B) in the outer layer of the skin of the seamless popping capsule makes the adsorbed water molecules diffuse unevenly in the skin, which produces a localized hardening phenomenon occurred in the process of 4 h to 6 h at 32 °C in Fig. 7.

### DIFFERENTIAL THERMODYNAMIC ANALYSIS

Thermodynamic properties are essential for the understanding of the stability of water-sensitive food products during storage and their shelf-life^13^. From accurate experimental adsorption isotherms and robust mathematical models, thermodynamic properties (Gibbs free energy, differential enthalpy and entropy, net isosteric heat of adsorption) can also be computed. Consequently, the thermodynamic analysis of sorption data provides a reliable perspective into the interaction between water molecules and the solid matrix^20^.

#### Gibbs free energy change

**Fig. 11:**
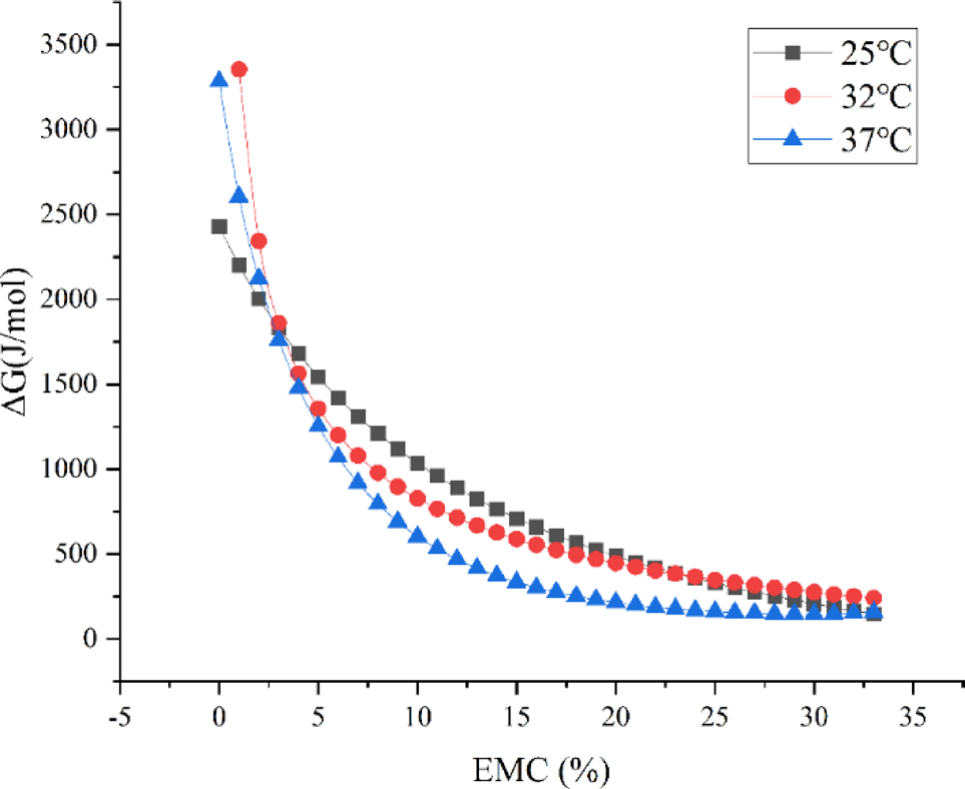
Gibbs free energy change of seamless popping capsules shell.

The variation of Gibbs free energy (ΔG) with equilibrium moisture content (EMC) during the adsorption process of the capsules at three different temperatures were shown in Fig. 11. A positive *ΔG* indicates that the water adsorption reaction is non-spontaneous and requires energy absorption from the surrounding environment, while a negative *ΔG* suggests a spontaneous reaction^12^. Evidently, *ΔG* decreases with increasing EMC, and all values of *ΔG* are positive, indicating that water adsorption in this system is not a spontaneous process. A high *ΔG* reflects the high degree of freedom for water adsorption due to the hydrophilicity of the food material. Indeed, in practical situations, i.e., when EMC exceeds 2.5%, the higher the temperature, the lower the *ΔG* at a specific EMC. *ΔG* is related to the energy required to make adsorption sites available, thus it decreases with increasing temperature or EMC. At higher EMC, the rate of increase in *ΔG* for the adsorbent becomes very slow. This is consistent with the report by Yogendrarajah et al^14^. Finally, the *ΔG* curves at different temperatures converge towards a single value, gradually approaching the Gibbs free energy at the isokinetic temperature (ΔG_*β*_).

#### Net isosteric heat of adsorption and differential entropy

**Fig. 12:**
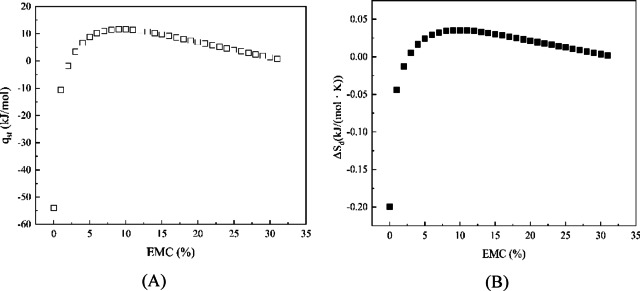
Net isosteric heat (**A**) of adsorption and differential entropy change (**B**) of seamless popping capsules shell.

The net isosteric heat of sorption (*q*_*st*_) is the difference between the sorption heat of a solid and the latent heat of evaporation of pure water, reflecting the interaction force between water molecules and the adsorption sites of the materials. When adsorption reaches equilibrium, it is approximately equal to the differential enthalpy^12^. Based on the best-fit isothermal adsorption models (Smith at 25 °C, GAB at 32 °C, and Smith at 37 °C), the relationship curves between the *q*_*st*_ of adsorption, differential entropy (*ΔS*_*d*_), and the moisture change rate at adsorption equilibrium (*EMC*) are shown in Fig. 12. As moisture increased, the *q*_*st*_ initially increased and then decreased, with a maximum value of 11.665 kJ/mol, which was less than the latent heat of evaporation of pure water (44 kJ/mol), indicating that water molecules existed in a free state within the shell of the seamless popping capsules. This trend was consistent with that observed in chia seed mucilage^13^ and polymer materials^21^, further corroborating the infrared analysis results. Similarly, in practical situations, the initial stage of moisture adsorption indicated that water molecules were preferentially bound to favorable adsorption sites on the capsule shell surface with a tight and high energy bonding, leading to an increase in enthalpy. The maximum enthalpy point represented the coverage of strong binding sites and the maximization of water-capsule shell interactions. Subsequently, as the favorable sites were covered and multilayer water molecules formed, the enthalpy decreased. Such differential enthalpy data were particularly valuable for estimating the energy required to reduce the moisture content of a food product from a given initial moisture content to a specific final moisture content^13^.

Differential entropy (*ΔS*_*d*_) is a crucial parameter for measuring the change in the number of adsorption sites in a material during the water adsorption process. At a specific energy level, its magnitude is proportional to the number of water adsorption sites per unit area of the material. In the seamless popping capsule system, the trend of entropy change was similar to that of enthalpy, characterized by an initial increase followed by a decrease, and there was almost no temperature inverse effect (*ΔS*_*d*_ < 0) in practical applications^20^. Combined with ATR-FTIR analysis results, the main water adsorption sites were the alcoholic hydroxyl groups at 3273 cm^−1^ (O–H stretching vibration). The exposure of a large number of these groups lead to an increase in differential entropy, making them favorable sites for water molecule adsorption. Additionally, the adsorbed water molecules gained mobility, increasing randomness, which also contributed to the increase in entropy. As adsorption progresses and the favorable sites become saturated, water molecules began to penetrate from the surface of the capsule shell. The cross-linked polymer molecules also restricted the rotational freedom and randomness of water molecules, resulting in a decrease in entropy. This demonstrated that the moisture absorption of the seamless popping capsule shell involved a pre-wetting stage, corroborating the foaming phenomenon observed in the cross-sectional SEM images.

#### Spreading pressure

**Fig. 13:**
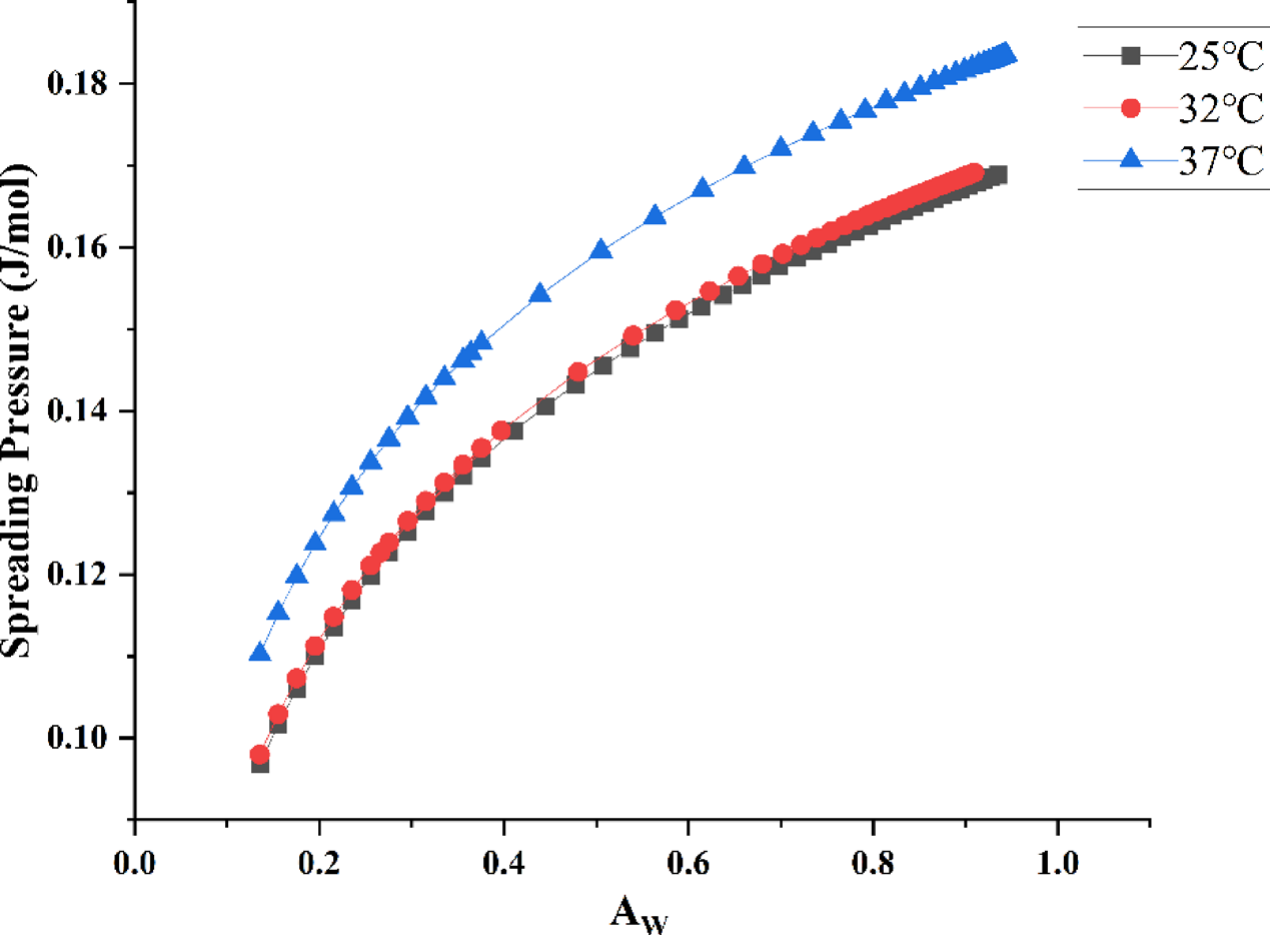
Spreading pressure isotherm of seamless popping capsules.

Spreading pressure is a measure of the increased surface tension at adsorption sites due to adsorption mechanisms, quantifying excess surface free energy^23,33^. Across the three experimental temperatures, it was observed that spreading pressure increases with rising *A*_*w*_ and escalates further at higher temperature (Fig. 13). This phenomenon likely stemmed from thermal-induced cracking in the gelatin molecular layer, which disrupted its adsorption interaction with water molecules. At elevated temperatures, water molecules penetrated the gelatin layer via permeation, leading to a sharp increase in surface tension.

#### The analysis of enthalpy–entropy compensation theory

**Fig. 14:**
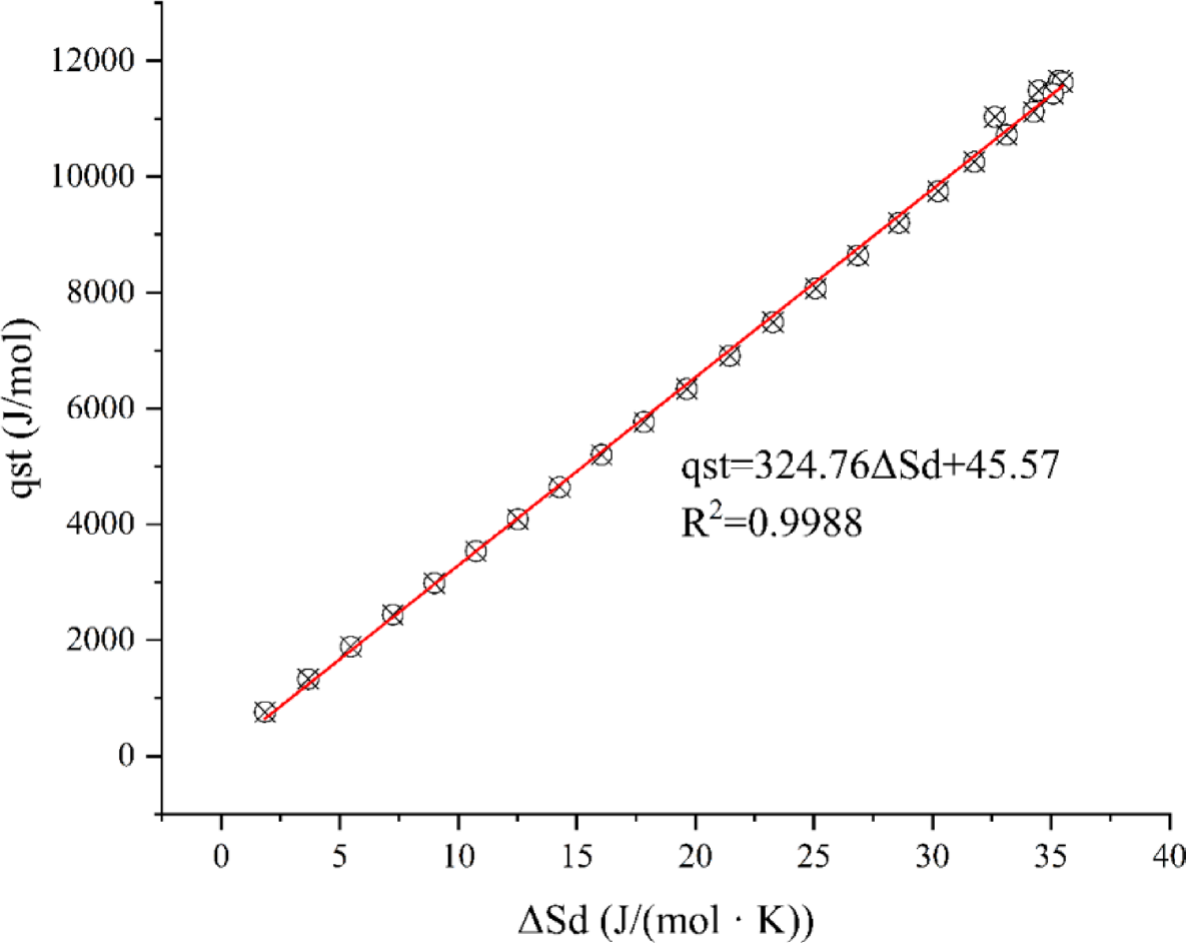
Relationship between the net isosteric heat of adsorption and differential entropy in the hygroscopic process of seamless popping capsules.

The enthalpy-entropy compensation theory reflects the physicochemical phenomena of water adsorption and is crucial for verifying the scientific validity of water adsorption experiments, holding significant importance for studying moisture adsorption in materials. As shown in Fig. 14, a strong linear relationship (R^2^ > 0.998) was observed between the differential entropy and the isosteric heat of adsorption, with *T*_*β*_ = 324.76 K, which was higher than the harmonic temperature *T*_*hm*_ (304.40 K). Therefore, the enthalpy-entropy compensation theory was applicable. Since *T*_*β*_ > *T*_*hm*_, the moisture adsorption process of the seamless popping capsules was enthalpy-driven and non-spontaneous (ΔG_*β*_ > 0). Consequently, by controlling the energy intensity of the storage environment, various undesirable phenomena of the popping capsule products, such as adhesion and decrease in the popping or melting mouthfeel, can be mitigated. It is worth mentioning that although ΔG_*β*_ > 0, its value was quite small, indicating that the moisture adsorption reaction in the seamless popping capsule system occurs easily. This further confirmed the need for strict control of the temperature and humidity of the storage environment and highlights the high requirements for the packaging of such products, as mentioned previously.

## Conclusion

The seamless popping capsules were stored under controlled conditions at 25 °C, 32 °C, and 37 °C, each with a relative humidity (RH) of 68%. Capsules stored at 37 °C and above lost their commercial viability due to structural and textural changes. Moisture adsorption followed an S-shaped pattern, characteristic of Type II isotherms, indicating a multilayer, reversible adsorption process. The adsorption data aligned well with the Smith model at 25°C and 37 °C (R^2^ = 0.9965 and 0.9827, respectively) and with the GAB model at 32°C (R^2^ = 0.9925). Findings suggest that storage humidity should not exceed 61% RH. Moisture sorption followed first-order kinetics (R^2^ > 0.95), with adsorption saturation occurring within 2 h. Spectroscopic analysis (ATR-FTIR) identified alcohol hydroxyl groups at 3273 cm^−1^ as primary moisture-binding sites. Microscopic imaging (SEM) revealed structural degradation under high temperature and humidity, including cracks, agglomeration, wrinkles, and surface bubbling. These structural changes were directly linked to moisture penetration, which affected the texture and sensory properties of the capsules. Thermodynamic analysis provided further insight into the moisture adsorption mechanism. The moisture adsorption process in seamless popping capsules involves initial surface interaction with water molecules, followed by multilayer adsorption driven by hydrophilic functional groups. Temperature and humidity significantly influence the adsorption capacity and rate, leading to structural degradation and changes in texture. As equilibrium moisture content increased, Gibbs free energy, net isosteric heat of sorption, and differential entropy decreased, eventually approaching zero at high moisture levels. The moisture sorption process followed the entropy-enthalpy compensation theory, with an isokinetic temperature (T_β_) exceeding the harmonic temperature (*T*_*hm*_) and ΔG_*β*_ remaining positive. These findings confirm that moisture adsorption in the capsules is an enthalpy-driven, non-spontaneous process. This study offers theoretical guidance for optimizing storage conditions and improving the stability of seamless popping capsules, ensuring their quality and commercial viability.

## Data Availability

The authors declare that the data supporting the findings of this study are available within the paper. Should any raw data files be needed in another format they are available from the first author upon reasonable request.
